# Current Status and Future Prospects for Esophageal Cancer

**DOI:** 10.3390/cancers15030765

**Published:** 2023-01-26

**Authors:** Mahdi Sheikh, Gholamreza Roshandel, Valerie McCormack, Reza Malekzadeh

**Affiliations:** 1 Genomic Epidemiology Branch, International Agency for Research on Cancer (IARC/WHO), 69007 Lyon, France; 2 Golestan Research Center of Gastroenterology and Hepatology, Golestan University of Medical Sciences, Gorgan 49341-74515, Iran; 3 Environment and Lifestyle Epidemiology Branch, International Agency for Research on Cancer (IARC/WHO), 69007 Lyon, France; 4 Digestive Oncology Research Center, Digestive Diseases Research Institute, Tehran University of Medical Sciences, Tehran 14117-13135, Iran

**Keywords:** adenocarcinoma, epidemiology, prevention, risk factors, squamous cell carcinoma

## Abstract

**Simple Summary:**

Over 500,000 individuals died due to esophageal cancer (EC) worldwide in 2020. More than four decades of etiological research have predominantly focused on the esophageal squamous cell carcinoma (ESCC) subtype, which accounts for 80% of EC cases, while less etiologic research has been done on the esophageal adenocarcinoma (EAC) subtype whose incidence rates are rapidly increasing in some high-income countries. Recent advances in genomics and microbiome studies, and the establishment of high-quality research infrastructure in high-risk regions, including Africa and Iran, have advanced our understanding of the causes and potential risk factors of these diseases in different regions. For therapy and early detection, topical studies that have used chemoprevention methods, such as using aspirin and proton pump inhibitors, and early detection methods, such as using Cytosponge™ and biomarker assays among symptomatic or high-risk individuals, have shown promising results. However, there are still challenges in each field that need to be addressed in future studies.

**Abstract:**

Esophageal cancer (EC) is the ninth most common cancer and the sixth leading cause of cancer deaths worldwide. Esophageal squamous cell carcinoma (ESCC) and esophageal adenocarcinoma (EAC) are the two main histological subtypes with distinct epidemiological and clinical features. While the global incidence of ESCC is declining, the incidence of EAC is increasing in many countries. Decades of epidemiologic research have identified distinct environmental exposures for ESCC and EAC subtypes. Recent advances in understanding the genomic aspects of EC have advanced our understanding of EC causes and led to using specific genomic alterations in EC tumors as biomarkers for early diagnosis, treatment, and prognosis of this cancer. Nevertheless, the prognosis of EC is still poor, with a five-year survival rate of less than 20%. Currently, there are significant challenges for early detection and secondary prevention for both ESCC and EAC subtypes, but Cytosponge™ is shifting this position for EAC. Primary prevention remains the preferred strategy for reducing the global burden of EC. In this review, we will summarize recent advances, current status, and future prospects of the studies related to epidemiology, time trends, environmental risk factors, prevention, early diagnosis, and treatment for both EC subtypes.

## 1. Introduction

Esophageal cancer (EC) is the ninth most common cancer and the sixth leading cause of cancer deaths worldwide [1]. It is a highly fatal disease, accounting for more than 500,000 deaths each year [2,3]. Esophageal squamous cell carcinoma (ESCC) and esophageal adenocarcinoma (EAC) are the two main EC histological subtypes that have distinct epidemiological and clinical features. Globally, ESCC is the most common EC subtype, which accounts for 80% of EC cases, and it can anatomically develop throughout the esophagus [4]. On the other hand, EAC (20%) is the most common subtype in white populations of developed countries, and it tends to develop in the distal esophagus [4]. While the global incidence of ESCC is declining, a rapid and consistent increase in the incidence of EAC in western countries has been reported in the past 40 years [4,5].

Decades of epidemiologic research have identified distinct environmental exposures that are the risk factors for either ESCC or EAC subtypes. Furthermore, recent advances in understanding the genomic aspects of EC have led to using specific genomic alterations in EC tumors as biomarkers for early diagnosis, treatment, and prognosis of this cancer [4].

Despite the improvements in EC survival rates in the past two decades, particularly among younger patients with EAC, the prognosis of EC is still poor, with a five-year survival rate of less than 20% [6,7]. Currently, there are significant challenges to early detection and secondary prevention for both ESCC and EAC subtypes, but Cytosponge™ improves early detection of EAC. Consequently, primary prevention remains the preferred strategy for reducing the global burden of EC. In this review, we will summarize epidemiology, time trends, environmental risk factors, prevention, early diagnosis, and treatment for both EC subtypes based on the latest research studies.

## 2. Epidemiology of Esophageal Cancer

*Overall incidence rates:* According to the Global Cancer Observatory (Globocan), in 2020, an estimated 604,100 individuals worldwide were diagnosed with EC (3.1% of all incident cancers), leading to crude rates of 7.8, an age-standardized incidence rate (ASR) of 6.3 per 100,000 person-years, and a cumulative risk (0–74 years) of 1.52% for this cancer [1,3]. Because prognosis is generally poor, a higher percentage (5.5%) of all cancer-related deaths were due to EC (544,000 deaths) than to new cases (3.1%) [3]. 

**Figure 1:**
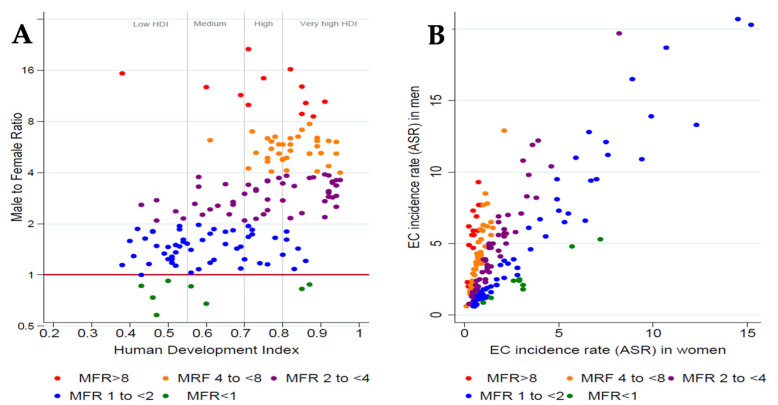
(**A**) Country-level male-to-female ratio (MFR) for esophageal cancer (EC) versus a country’s Human Development Index. (**B**) Country-level age-standardized incidence rates (ASR) for esophageal cancer among men versus rates among women. Both plots are color-coded according to categories of the MFR as indicated. Data source: Globocan 2020.

*Incidence by gender:* Globally and in most settings, EC is more common in men than women. According to the Globocan 2020 estimates, 69% of new EC cases occurred in men. ASRs were 2.5-fold higher in men (9.3) than women (3.6), with notable variations in a male-to-female ratio (MFR) between populations. The highest MFRs were reported for countries with very high development index (HDI) (Figure 1A), which occurred when ASRs were extremely low in women (Figure 1B). The lowest MFR was reported for countries with low HDI (Figure 1A), but these ratios include the full ranges of ASR. The countries with the highest ASRs for EC tend to fall in this range, i.e., with moderate relative differences in ASRs but large absolute differences in ASRs (Figure 1B) [1,3]. However, a female excess in the ASR of EC has also been reported in a few instances, e.g., in the 1970s in North-Eastern Iran and countries indicated with green dots in Figure 1B, which include Ethiopia, Somalia, and Yemen [8].

**Figure 2:**
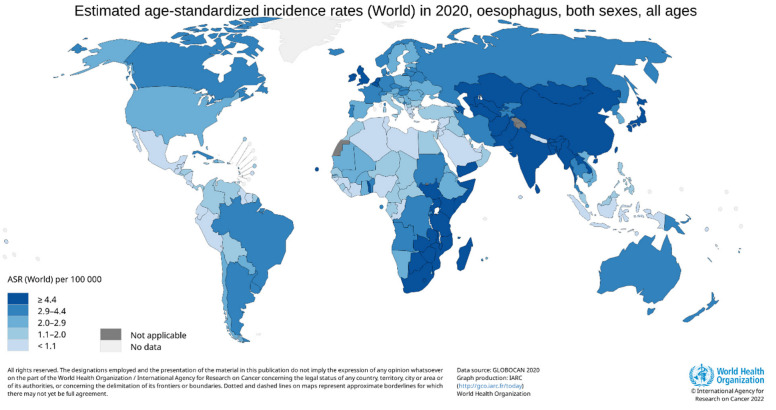
High incidence areas of esophageal cancer (EC) in Eastern and South-Central Asia (Asian EC belt) and Eastern and Sothern Africa (African EC corridor).

*Incidence by population:* There are large variations in EC incidence rates between different populations [5,9,10,11]. In the 1970s, an extraordinarily high incidence rate of esophageal cancer (>100 per 100,000 person-years) was reported from North-Eastern Iran and Linxian in China [8,12]. In 2020, at a country level, the highest ASRs for EC were estimated for Malawi (ASR 17.5 per 100,000 person-years) and Mongolia (17.1), and the lowest rates were estimated for Belize (0.28) and Congo (0.33). Considering world regions, Eastern Asia (12.3), Eastern Africa (7.3), Southern Africa (6.7), and South-Central Asia (5.6) exhibit the highest ASR for EC, while the lowest rates were reported for Central America (0.93) and Western Africa (1.3). The high-incidence rate areas in Asia (including Eastern Asia and South-Central Asia) are traditionally known as the Asian esophageal cancer belt and those of Africa (including Eastern Africa and Southern Africa) as the African esophageal cancer corridor. About 80% of EC cases worldwide occur in these two high-risk regions, which are predominated by ESCC (Figure 2) [1,3,5,7].

*Incidence by histological type:* As already stated, the two major histologic subtypes of EC are ESCC and EAC. Of the total 604,100 new EC cases in 2020, 85,672 (14%) were EAC with an ASR of 0.9 per 100,000 person-years, and 51,2469 (85%) were ESCC with an ASR of 5.4 per 100,000 person-years [13]. The ASR of ESCC were 7.8 and 3.2 Per 100,000 person-years, while those reported for EAC were 1.4 and 0.4 in men and women, respectively [13]. In 2018, the highest ASR of ESCC occurred in Eastern Asia (11.1 per 100,000 person-years), South-Central Asia (4.8), and Sub-Saharan Africa (4.2), i.e., in the Asian belt and African corridor, and the highest ASR of EAC, although much lower than the highest for ESCC, were reported from Northern Europe (3.5), Northern America (2.2), and Oceania (2.0). ESCC is the dominant subtype of EC in most countries and at the global level, but in some highly developed countries, including Australia, Canada, the United States, and Northern Europe countries, the incidence of EAC was higher than ESCC [13,14,15,16] by 2020.

*Incidence trends over time:* Recent reports suggest a global decreasing trend in EC incidence rates [5,17,18,19,20,21]. The global ASR of EC decreased by 22% during the last three decades [9,22,23]. However, there is heterogeneity in time trends across populations [5,24,25]. A decreasing trend is reported in countries that formerly had a high-risk population for EC, including most Asian countries [26,27,28], while in low-risk populations, including the United Kingdom, the Netherlands, and the United States, an increasing trend in the incidence of EC has been observed [29,30,31,32]. These differences may partly be due to the opposing trends by EC histological subtypes [5,30]. Recent reports indicate decreasing incidence trends for ESCC and increasing incidence trends for EAC [5,33]. Therefore, in countries with a high proportion of ESCC (most Asian countries), the decreasing trends in EC incidence rate are mostly due to the reduction in ESCC rates [5,26,27,28]. In contrast, the observed increasing trends in EC incidence rates in countries with a high proportion of EAC (e.g., the United Kingdom and the Netherlands) are predominantly due to an increase in the incidence rate of EAC, most notably in men [5,33].

*Future burden:* The incidence rate and absolute numbers of EAC are predicted to considerably increase across high-income countries, and the incidence rate of ESCC is predicted to consistently decrease in almost all populations (especially in those with high ESCC rates) during the next few decades [25,26,27,30,32,33]. The ASR of EAC is predicted to increase from 6.33 per 10^5^ person-years in 2005 to 7.76 in 2030 in the United Kingdom and from 5.32 in 2005 to 8.65 in 2030 in the Netherlands [33]. On the other hand, the ASR of ESCC is predicted to decrease from 5.34 per 10^5^ person-years in 2005 to 1.54 in 2030 in France and from 4.94 in 2005 to 1.57 in 2030 in the black population of the United States [33]. Due to these time trends, the incidence of EAC has surpassed (in high-income countries) or is predicted to surpass the incidence of ESCC in some populations in the next decades [33]. For the African EC corridor, high-quality temporal data are less abundant, and the few available long-term trend data do not indicate substantial declines in ESCC incidence rates.

## 3. Environmental Risk Factors for Esophageal Cancer

As mentioned earlier, ESCC and EAC have distinct risk factors. ESCC is a multifactorial disease with a long list of suspected risk factors, whereas EAC has much fewer well-established risk factors. Below we provide an overview of these risk factors and risk markers which are also summarized in Table 1.

### 3.1. Habits

#### 3.1.1. Tobacco

The International Agency for Research on Cancer (IARC) monograph program has classified tobacco smoking and the use of smokeless tobacco as a cause of EC [34]. In 2019, an estimated 203,000 EC deaths worldwide (51% of EC deaths in men and 12% in women) were attributable to tobacco smoking [35]. While both EC subtypes are linked to tobacco smoking, the relationship is stronger for ESCC than EAC [36].

The magnitude of the relative risk estimates for smoking tobacco and ESCC risk vary largely between populations, with three- to nine-fold increased risks in more developed countries (low-risk areas) [37,38,39], two- to eight-fold risk increase in Sub-Saharan Africa [40,41], and a null to a minimum (30–50%) risk increase in Asia [42,43,44,45,46]. A recent analysis of 552 ESCC genomes from eight countries with varying incidence rates showed broadly similar average relative contributions of tobacco-related mutational signatures between ESCC cases from high-risk and low-risk regions [47]. Altogether, these findings confirm a causal role for tobacco smoking in ESCC and suggest that the lower risk estimates in epidemiologic studies from high-risk regions might reflect the exposure to other strong risk factors that account for most ESCC cases, which could dilute the effect of tobacco smoking [48]. Differences may also be due to effect modification by third factors or differences in pack-years as the number of cigarettes smoked per day often varies between populations.

Other tobacco exposure routes and modes of use, such as waterpipe smoking [49,50], using smokeless tobacco (chewed or placed in the nose or under the tongue) [43,51], and chewing betel quid (containing areca nut, betel leaf, tobacco, and variety of ingredients [52], have been associated with around three-fold increased ESCC risk [53,54]. 

Studies have reported a two- to three-fold increase in EAC risk among current smokers vs. never smokers [55,56,57]. An estimated 38% of EAC cases in the United States are attributable to smoking cigarettes [55].

#### 3.1.2. Alcohol

The IARC Monograph program has classified alcohol consumption as a cause of EC [58]. In 2019, an estimated 114,000 esophageal cancer deaths worldwide (28% of EC deaths in men and 7% in women) were attributable to alcohol drinking [35]. Alcohol drinking increases the risk of ESCC but not EAC [55,59,60,61].

A recent meta-analysis of 74 studies (18 cohort and 56 case-control) showed a five-fold increased ESCC risk for the highest versus lowest alcohol intake [60]. This meta-analysis also indicated a linear dose-dependent increase in ESCC risk with alcohol consumption (33% risk increase for every 12.5 g/day increase in alcohol intake) [60]. Variations between studies are likely to reflect the challenges in the assessment of lifetime ethanol intake, especially in settings where there is frequent consumption of locally produced fermentations and distillations in rural areas, where this disease is more common; these alcohols may never appear in official statistics; some are illegal, and even if reported, their ethanol concentrations can be highly variable. The reported population-attributable fractions of ESCC due to alcohol consumption vary largely between populations, with around 40% of cases (concentrated among men) in Sub-Saharan Africa [62] and around 20% of cases in economically-developed countries [35,55] being attributed to alcohol consumption, while no major role has been identified for alcohol consumption for ESCC in Asia (particularly in high-risk regions) [42,43,46]. Consistently, a recent analysis of 552 ESCC tumor genomes from eight countries with varying incidence rates showed significant between-country differences in the presence of alcohol-related signatures, which were present in ESCC tumors from Sub-Saharan Africa, Japan, and the United Kingdom, but not in cases from Iran and China [47]. 

#### 3.1.3. Opium

While the IARC Monograph program has classified opium consumption as carcinogenic to humans, the evaluation in 2020 indicated that there was limited evidence on whether opium use can cause EC [63]. The literature on opium use and EC risk exclusively focused on the ESCC subtype, and it almost all originated from Iran, where 40% of the world’s opium is consumed [63]. These studies found a 1.6- to 2-fold increased ESCC risk among opium users versus never users [43,46,64,65]. Furthermore, a recent analysis of ESCC tumor genomes from 178 cases from Iran identified a specific mutational signature that could potentially be driven by opium exposure [47].

#### 3.1.4. Hot Food and Drinks

In 2016, the IARC monograph program classified drinking very hot beverages, defined as ≥65 °C, as “probably carcinogenic to humans” (known as Group 2A) [66]. Drinking hot beverages has consistently been found to increase ESCC but not EAC risk [66,67]. Several meta-analyses showed more than two-fold increased ESCC risk for drinking beverages at higher versus lower temperatures [67,68,69,70]. Despite the differences in the beverage types (green tea, salt tea, black tea, coffee, and mate), when drank at high temperatures, they were frequently linked to increased ESCC risk [40,71,72,73,74,75,76,77,78,79,80]. While the majority of the available studies estimated thermal exposures during the consumption of hot beverages by self-reported perceptions that may vary across individuals [66,67], several studies assessed ESCC risk in relation to objectively measured drinking tea temperature at first sip and found around two-fold increased ESCC risk among those who drink tea at ≥60 °C versus <60 °C [71,81,82]. However, a single temperature at the first sip of a drinking episode is a limited measurement of the entire thermal exposure experienced during hot beverage consumption, which has previously been shown to depend on both the sip volume and temperature throughout the drinking episode that occurs at a certain speed which influences the time left for the liquid to cool [83]. Consuming hot water and hot food (including porridge and soup) has also been linked to higher (over four-fold) ESCC risk in a dose-response manner assessed using a 12-point thermal exposure index [72,82]. Expansion of comprehensive measurement studies of intra-esophageal thermal exposures during hot food/beverages consumption is needed, covering both high and low ESCC risk settings, habits across the life course, mechanistic pathways, and possible effect modifications. 

### 3.2. Living Environment

#### 3.2.1. Socioeconomic Status (SES)

While incidence rates of ESCC are higher in low- and middle-income countries and the converse holds for EAC, in within-country analyses, low SES is associated with a 1.5 to 2-fold increased risk of both ESCC and EAC subtypes [43,61,76,84,85,86,87,87,88,89]. SES is a complex concept and has traditionally been defined by education, wealth, and occupation [90]. It is a marker of risk rather than a risk factor. While the exact mechanisms for these associations are unclear, it is likely an accumulation of biological risk factors with strong social gradients, which cannot be precisely measured or are unknown. These include exposure to damaging and potentially carcinogenic agents in the environment (e.g., in water or biomass fuel exposures), higher prevalence of risky behaviors and unhealthy lifestyles (e.g., poorer nutrition and oral health, and residual confounding by alcohol and tobacco), and higher exposure to the biological effects of living and working in a stressful environment that is linked to lower SES [90].

#### 3.2.2. Household Fuel

The IARC monograph program has classified household combustion of biomass fuel as probably carcinogenic to humans [91]. Studies on using biomass and kerosene fuels and EC risk are limited to the ESCC subtype. Recent studies from Iran [92], and Sub-Saharan Africa [80,93,94,95], showed around a two- to three-fold increase in ESCC risk when using biomass versus gas for household purposes. Studies also showed that among those who use biomass fuels, ESCC risk is higher with using non-chimney versus chimney-equipped stoves [92] and with cooking in the sleeping room versus in a separate room [74]. Few studies also found associations between using kerosene versus gas and higher risk for ESCC [92,94]. Whilst this evidence has amounted, exposure assessment has been limited to binary dichotomizations with a simple comparison of users vs. non-users of biomass fuels, i.e., two population groups that differ greatly by SES, and thus, confounding may be present. Studies with improved exposure assessment are needed. Further, as climate action promotes the use of clean fuels, mechanistic and epidemiologic studies to assess whether there are any gains in lowering ESCC risk are needed.

#### 3.2.3. Water Source

Studies on drinking water sources and EC risk are limited to the ESCC subtype. Drinking un-piped (untreated) water from different sources, including wells, cisterns, and rivers, has been linked to around a two-fold increase in ESCC risk in different regions, including China [42,96], Iran [43,97], and Sub-Saharan Africa [94,98]. While drinking un-piped water might be confounded by risk factors that also have a higher prevalence among people with low SES, the association with ESCC in neighborhood/residence matched controls [97], and the dose-dependent relationship [43,97] indicates that drinking untreated water could be a potential route of exposure to different carcinogens, such as nitrates, phosphates, heavy metals, or water properties (e.g., alkalinity) [99]. Higher concentrations of certain chemical compounds (e.g., nitrates, sulfate, fluoride) and lower concentrations of certain trace elements in drinking water from high-risk versus low-risk areas have been seen in ecologic studies from Iran [100], China [101,102], and Sub-Saharan Africa [98]. Studies of ESCC risk that can incorporate household-level water sampling are warranted in settings where the partial or full-time use of un-piped water is frequent. 

#### 3.2.4. Mycotoxin Contamination

Mycotoxins are naturally occurring secondary metabolites of several toxigenic fungi which can grow on different foods [103]. Fumonisins (classified as probably carcinogenic to humans by IARC monographs (Group 2B)) and aflatoxins (classified as carcinogenic to humans by IARC, with their impact on liver cancer, not EC) are the main mycotoxins subtypes that have been linked to EC [103]. Studies on mycotoxins are limited to the ESCC subtype.

Ecologic studies from China [104,105,106], Iran [107,108], and Africa [109,110] have shown higher exposure to fumonisins in high-incidence compared to low-incidence areas. Few ecological studies also found high levels of exposure to aflatoxins in high-risk areas of Iran and Africa [111,112]. However, data from observational epidemiologic studies are scarce and have shown inconsistent results so far [40,113,114,115]. 

#### 3.2.5. Animal Contact

Two case-control studies in Iran [116] and India [117] reported daily contact with animals to be associated with increased ESCC risk. However, a recent prospective cohort analysis from Iran did not confirm this association [43]. Additionally, a recent study measured immunoglobulin G anti-Coxiella burnetii and anti-Brucella spp. antibodies in 177 ESCC patients and 177 residence-matched population-based controls, which did not find differences in the measured antibodies [118]. This might indicate that animal contact is confounded by the other risk factors, which are also associated with SES [116,117].

### 3.3. Individual Health 

#### 3.3.1. Gastroesophageal Reflux Disease (GERD)

Symptomatic GERD is the strongest known risk factor for EAC and its precursor lesion Barrett’s esophagus [48,119,120]. A pooled analysis of 1128 EAC cases and 4057 controls showed a dose-dependent association between the frequency and duration of GERD symptoms and EAC risk, reaching a nine-fold increased EAC risk among individuals who have had weekly GERD symptoms for ≥20 years compared to the controls [119]. This dose-dependent association has been frequently documented in studies from different geographic regions [121]. A recent study estimated that 20% of EAC cases in the United States could be attributed to GERD [55].

Although GERD was not previously recognized as a risk factor for ESCC [48], a prospective cohort study of almost 500,000 participants in the United States showed a two-fold increased ESCC risk among individuals with GERD during a 16-year follow-up period [122]. However, another prospective cohort study of 50,000 participants in a high-risk region of Iran showed no increase in ESCC risk among individuals with GERD during a 13-year follow-up period [123].

#### 3.3.2. Body Mass Index (BMI)

Studies on BMI and EC have consistently shown contrasting risk association patterns for ESCC and EAC subtypes. Higher BMI is frequently linked to lower ESCC and higher EAC risks. In a recent meta-analysis of 25 studies, in comparison to having a normal BMI, being underweight was associated with a 57% higher risk for ESCC, and having a higher BMI was associated with having a 27% to 37% lower risk for ESCC, and 56% with more than the two-fold higher risk for EAC [124]. A recent study showed that an estimated 15% of ESCC cases and 38% of EAC cases in the United States are attributable to low and high BMI, respectively [55]. The lower risk for ESCC does not seem to be explained by reverse causality as the inverse associations between BMI and ESCC risk has been shown to exist a decade or more prior to the diagnosis of ESCC [125,126,127].

#### 3.3.3. Gastric Atrophy

A meta-analysis of nine studies showed that patients with gastric atrophy have almost a two-fold increased risk for ESCC and no risk for EAC [128]. The results were similar in the subgroup of studies from high-risk (China) and low-risk areas (Europe and Japan) [128]. The increased ESCC risk was confirmed in later case-control studies from high-risk areas, including Iran [129], Latin America [130], and China [131]. A recent meta-analysis of eight studies (five case-control and three prospective cohorts) assessed the relationship between serum pepsinogens, which are serological biomarkers for gastric atrophy, and ESCC risk, which similarly showed almost a two-fold increased ESCC risk with lowest vs. highest levels of serum pepsinogen I [132].

#### 3.3.4. Poor oral Health and Hygiene

Different indicators of poor oral health and oral hygiene, including tooth loss, an index of the sum of decayed, missing, and filled teeth (DMFT), and less frequent or no tooth brushing, have been linked to increased ESCC risk in high-risk regions [16,43,80,98,133,134,135,136,137,138,139], while inconsistent results (for both ESCC and EAC subtypes) were obtained from studies in low-risk regions [140,141,142,143]. A recent prospective cohort of five million individuals who underwent dental examinations in Sweden found an increased risk for both ESCC and EAC subtypes with increasing tooth loss and periodontitis at baseline [144]. Meta-analyses indicate more than two-fold increased EC risk in the lowest vs. highest frequency of tooth brushing and a 20% to 30% increase in EC risk in the highest vs. lowest categories of the tooth loss [145,146,147,148]. Mechanistic studies are needed to help understand these relationships, which might include dysbiosis of the correlated oral and esophageal microbiomes, as explained below.

#### 3.3.5. Microbiome

Although the studies on microbiome and EC are at their early stages, they indicate a potential role for the oral and esophageal microbiome in the risk of ESCC and EAC [149]. Several studies, mostly cross-sectional with a limited number of cases, assessed oral or esophageal microbiome in relation to ESCC, esophageal squamous dysplasia (ESD, precursor lesion for ESCC) [150,151,152,153,154,155,156,157], EAC, or Barrett’s esophagus (precursor lesion for EAC) [158,159,160,161,162]. Altogether, these studies indicate that the composition of oral bacterial communities (e.g., an abundance of Prevotella species) and esophageal bacterial communities (e.g., an abundance of Fusobacterium and Clostridiales species), lower oral microbial diversity, and lower esophageal microbial richness may be linked to ESD and ESCC [149,163], while changes in the ratio of Gram-negative esophageal bacteria (mainly Streptococcus and Prevotella) may be linked to Barrett’s esophagus [149]. No clear, consistent pattern has been observed for EAC in studies so far [149]. Future studies need to have prospective study designs because the oral and esophageal microbiomes of ESCC patients are likely to be critically altered due to the disease’s central and often severe symptom of dysphagia. 

### 3.4. Dietary Factors 

Numerous studies evaluated the associations between specific dietary groups or micronutrients and EC risk. Based on a recent umbrella review of meta-analyses, altogether, these studies indicate that habitually consuming calcium, zinc, whole grains, fruits, and vegetables is associated with a lower risk of EC [164]. However, these findings should be interpreted with caution as some associations were graded as weak, and most are derived from case-control studies with significant heterogeneity [164]. Because different dietary components are consumed in combination and because they usually correlate and interact with one another, recent recommendations suggest studying whole dietary patterns in relation to different health outcomes [165,166]. Nevertheless, for ESCC, the unusual geographical pattern of the high-risk areas points to a potential role of some geographical pattern risk factors, which might include diet in communities that rely upon subsistence farming. 

#### 3.4.1. Whole Diet Quality

Two prospective cohorts evaluated whole diet quality using the Healthy Eating Index (HEI) and Mediterranean diet (MED), which showed a dose-dependent decrease in ESCC risk with having a better diet quality (higher HEI and MED scores) [167,168]. Few case-control studies measured the dietary inflammatory index (DII) in relation to ESCC and showed that a pro-inflammatory diet (higher DII score) is associated with increased ESCC risk [169,170]. The available dietary scores are derived from studies in high-income countries, and limited or no data are available on diet quality and risk of ESCC from high-risk areas that have different dietary patterns.

EAC risk did not show a significant relationship with adherence to the MED diet, which has a plant-based food foundation [167,168], while inverse non-dose dependent association was also reported for EAC risk and HEI [167]. Lower EAC risk is frequently linked to more adherence to the World Cancer Research Fund (WCRF) and the American Institute for Cancer Research (AICR) (WCRF/AICR) recommendations that include dietary, physical activity, and BMI components [61,171,172,173].

#### 3.4.2. Intake of Specific Dietary Groups

*Fruits and vegetables:* higher intake of fruits and vegetables is recognized by IARC to probably lower EC risk [174]. A recent study estimated 51,000 and 17,000 EC deaths worldwide to be attributable to low consumption of fruits and vegetables, respectively [35]. Two meta-analyses (32 studies for ESCC and 12 studies for EAC) found around 45% and 25% lower risk for ESCC and EAC, respectively, for the highest vs. the lowest intake of fruits and vegetables [175,176]. 

*Pickled vegetables:* pickled vegetables are an integral part of the Chinese diet [177]. The pickling process could generate some carcinogenic compounds, including nitrosamines, mycotoxins, and PAHs [163,177]. IARC classifies pickled vegetables prepared traditionally in Asia as possibly carcinogenic to humans [178]. Since then, many studies (mainly case-control) have assessed the association between pickled vegetables and ESCC risk. Two meta-analyses of these studies showed a two-fold increase in ESCC risk for the highest vs. the lowest consumption of pickled vegetables/food [179,180].

*Red and processed meat:* meta-analyses show that the highest vs. the lowest intake of red meat and processed meat is associated with 30–70% higher risk for both ESCC and EAC subtypes [180,181,182,183,184,185]. A meta-analysis showed that the estimated risk increase is derived from case-control studies and was not found in cohort studies [186].

*Other dietary groups:* two recent meta-analyses showed a reverse relationship between consuming carbohydrates and risk for both ESCC and EAC [187,188]. The available meta-analyses on the intake of fish and poultry showed no effect on EAC risk, while the results on ESCC were inconsistent, ranging from null to protective effects [182,184,189,190]. Similarly, no associations were found between consuming dairy products and dietary fats with ESCC risk, while higher consumption of total fat, saturated fat, and monounsaturated fat has been linked to a higher EAC risk [191,192,193]. 

#### 3.4.3. Micronutrients

There have been numerous studies with inconsistent results on the relationship between different micronutrients or their biomarkers (in diet /serum /urine) and ESCC risk. In the 1980s, severe deficiencies in multiple vitamins and micronutrients were observed in a high-risk area in China (Linxian) [194], which resulted in initiating two interventional studies to assess the effects of multivitamin and micronutrient supplementation in the prevention of esophageal cancer in patients with esophageal squamous dysplasia [195], and in the general population [196]. The results of these two studies after 6, 10, and 25 years showed none to slight reduction (8%) in ESCC risk in one of the study arms (selenium, vitamin E, and beta-carotene), which was greater if supplementation was administered under age of 50 years [197,198]. However, a meta-analysis of interventional trials indicated no effects of vitamin and antioxidant supplements in preventing esophageal cancer [199]. 

Experimental, ecologic, and observational studies have implicated selenium and zinc deficiencies that might play roles in developing ESCC [200,201,202,203,204,205,206,207]. However, null or even contrasting results are also reported from some ecologic and observational studies in some high-risk areas [208,209,210,211,212]. In a recent meta-analysis, each 5 mg/day increase in zinc intake was associated with a 15% reduction in the EC risk [213].

### 3.5. Infections

#### 3.5.1. Viral Infections

*Human Papilloma Virus (HPV)*: after four decades of research, the role of HPV in the etiology of EC remains debated. In 2007, IARC concluded that there is inadequate evidence in humans for the carcinogenicity of HPV in the esophagus [214]. Since then, numerous studies with various designs and contradictory results have been conducted [215,216]. Some studies investigated HPV DNA in esophageal tumor samples, which reported varying prevalences ranging from 0% to 75% [216,217,218,219]. Other studies assessed the presence of serum antibodies against HPV and the risk of EC that provided contradictory results ranging from strong risk increase to protective effects [220]. Similarly, the results of the pooled and meta-analyses provided contradictory findings ranging from a limited effect [221] mild (around 60%) increase in ESCC risk [220] to a strong (three-fold) increase in ESCC risk [222,223]. Most notably, the large international Interscope study found that few HPV subtypes related to ESCC were present in very few cases, i.e., the population-attributable fraction would be very low [221]. However, a recent meta-analysis separately analyzed the risk of EC based on the study design and found that in case-control studies, the observed pooled risk was much higher (19 studies, around three-fold increased risk) than in cohort (11 studies, 20% increased risk, non-significant), and cross-sectional studies (three studies, 12% increased risk, non-significant) [224]. 

Other non-HPV viruses, including human immunodeficiency virus (HIV), Epstein–Barr virus (EBV), hepatitis B virus (HBV), and hepatitis C virus (HCV), have been less studied in relation to EC. However, current evidence suggests these viruses might play a minimum role, if any, in developing esophageal cancer [225,226,227,228]. A recent meta-analysis assessed the relationship between esophageal cancer and nine non-HPV virus types, which only showed a modest increase in esophageal cancer risk in relation to HBV and HCV infection, but not with HIV and EBV infections [225].

#### 3.5.2. Bacterial Infections

*Helicobacter pylori (H. pylori):* More than three decades of epidemiologic studies have frequently shown an inverse relationship between *H. pylori* infection and EAC risk. Although *H. pylori* infection can cause gastric atrophy [229], no consistent pattern was found for the relationship between *H. pylori* infections and ESCC [230,231,232,233]. Meta-analyses show that *H. pylori* infection is associated with around 50% decreased risk for EAC’s precursor lesion (Barrett’s esophagus) [234,235,236] and EAC [230,231,232,233].

## 4. Prevention

A recent analysis from the Global Burden of Disease study estimated that more than 68% of esophageal cancer deaths could be attributable to the identified environmental risk factors [35], which is consistent with findings from the local studies in high-risk [43] and low-risk regions [55]. Therefore, primary prevention is a critical step toward decreasing the burden of this disease worldwide [9]. At the population level, policymakers should implement primary prevention strategies that reduce exposure to the identified risk factors by improving basic social infrastructure and public education. Improvement of SES, population-health awareness and diet quality in high-risk regions were associated with a decline in the incidence of ESCC in these areas [5,85,237,238,239]. However, the rapid epidemic rise in the incidence of EAC [240,241] is concerning and warrants further investigation as this increasing trend could not be explained only by the increasing prevalence of well-known EAC risk factors, such as obesity, GERD, and tobacco smoking [5]. Research is needed to find preventable unidentified EAC risk factors to combat the rapid increase in this EC subtype.

Recent efforts were made to propose risk models based on different demographic profiles and exposure to certain risk factors to estimate the future risks for both ESCC and EAC subtypes to guide targeted screening in the general population [242,243]. However, most available EC risk prediction models have a high risk of bias and need further improvement in their quality and applicability [244,245].

**Figure 3:**
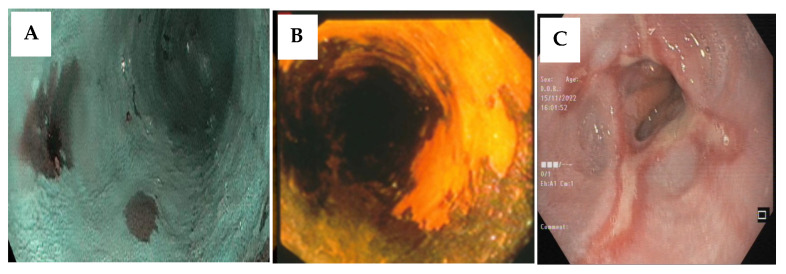
(**A**,**B**) Chromoendoscopy with acetic acid using narrow-band imaging and Lugol’s iodine staining both increase identification of HGD and early ESCC, which makes targeted biopsy for diagnosis and ablation or resection of HGD the precursor of ESCC possible. (**C**) Endoscopic image of Barrett’s esophagus visible as a salmon-colored metaplastic epithelium (columnar) replacing the normal bright-pink epithelium of the distal squamous esophagus.

During the last two decades, advances in endoscopic imaging and in resection and ablation techniques resulted in significant progress in the early detection and treatment of EC precancerous lesions and have paved the way for therapeutic interventions to prevent the progress of premalignant lesions toward cancer. Using upper gastrointestinal endoscopy, it is also possible to identify high-risk patients who would benefit from further surveillance. The precursor lesions for ESCC (high-grade squamous dysplasia, Figure 3A,B), and EAC (Barret’s metaplasia, Figure 3C), can be detected and removed using endoscopic techniques. Studies have shown that the removal of precursor lesions could prevent the development of cancer [246]. Currently, endoscopic eradication therapy (EET) is the standard of care for managing Barrett’s esophagus and early Barrett’s neoplasia. Endoscopic mucosal resection (EMR) and endoscopic submucosal dissection (ESD) are the two available resection techniques. After complete resection of all visible lesions, it is crucial to perform endoscopic ablation to ensure complete eradication of the remaining Barrett’s segment. Endoscopic ablation can be done with either thermal techniques, including radiofrequency ablation and argon plasma coagulation, or cryotherapy techniques. EET’s main goal is to completely remove intestinal metaplasia and prevent progression to dysplasia. Surveillance endoscopic examinations are necessary for early detection of recurrent Barret’s esophagus. Dysplastic Barret’s esophagus should be treated by expert endoscopists in special endoscopy centers. EET has advanced the field of Barret’s esophagus therapy and is currently the first-line therapy of choice for dysplastic Barret’s esophagus and intramucosal carcinoma with the aim of accomplishing complete eradication of intestinal metaplasia [247].

Thermal ablation techniques include radiofrequency ablation (RFA), argon plasma coagulator (APC), cryoablation techniques, liquid-nitrogen cryospray, and nitric-oxide cryoablation. Studies have shown the effectiveness of RFA in ablating dysplastic Barrett’s esophagus. A recent study reported the failure rate for RFA in patients with Barret’s esophagus to be up to 6% [248]. However, the treatment failure rate was reported to be as high as 40% in some studies [249]. Therefore, special training for using RFA is necessary for endoscopists. The incidence of EAC in patients with Barret’s esophagus following RFA is estimated to be about 1% [250].

Photodynamic therapy (PDT) is one of the longest-used ablation techniques in the treatment of Barret’s esophagus [251]. PDT has been shown to be an effective and safe method for eliminating dysplasia and preventing the development of esophageal cancer [252]. The advantages of PDT include selectively targeting the mucosal layer while minimizing structuring and perforation [252]. However, several limitations in the PDT method, including the need for intravenous administration agents and extended periods of photosensitization, have led to a decrease in using this method compared to RFA [252]. Finally, a study that compared the efficacy of PDT and RFA in treating Barret’s esophagus showed a higher histopathological remission for RFA (88.7%) than PDT (54.5%) [253]. 

Liquid nitrogen spray cryotherapy (LNSC) is a non-contact method for eradicating Barret’s esophagus that has been used both as primary treatment and salvage therapy. A recent meta-analysis of 14 studies with 707 Barret’s esophagus patients showed an overall pooled rate of complete eradication of 80.8% for dysplasia, 90.3% for high-grade dysplasia, and 55.8% for intestinal metaplasia with follow-up ranging from 4.25 months to 69.7 months [254]. The rates of the post-therapy strictures and perforation in LNSC are similar to those reported with RFA [254].

In addition to the mentioned interventions, chemoprevention approaches have also been investigated in patients with Barrett’s esophagus. By inhibiting cyclooxygenase (COX1 and COX2) and reducing prostaglandin secretion, aspirin is proposed to prevent the carcinogenesis of gastrointestinal cancers, including EAC. The esomeprazole and aspirin in Barrett’s esophagus (AspECT) study, a large phase-3 randomized prospective factorial study, assessed chemoprevention by aspirin and/or proton pump inhibitors (PPI) in patients with Barrett’s esophagus and found that a combination chemoprevention therapy with high-dose PPI and aspirin significantly and safely improved outcomes (reduce the risk for mortality and EAC) in patients with Barrett’s esophagus [255]. Combining a PPI with aspirin reduces the risk of gastrointestinal bleeding related to aspirin use. In the AspECT trial, only 1% of participants had a serious adverse event (mainly gastrointestinal bleeding). In this study, a high-dose (300 mg to 325 mg) daily aspirin was used, which is higher than the standard low dose of 75 mg that is commonly prescribed. Therefore, prescribing a low-dose aspirin with PPI might be associated even with a lower rate of adverse events than the 1% reported in the AspECT trial [255]. The number needed to treat, calculated in the AspECT study to prevent high-grade dysplasia, adenocarcinoma, or death in patients with Barrett’s esophagus, was 43 in the aspirin group [255,256].

**Figure 4:**
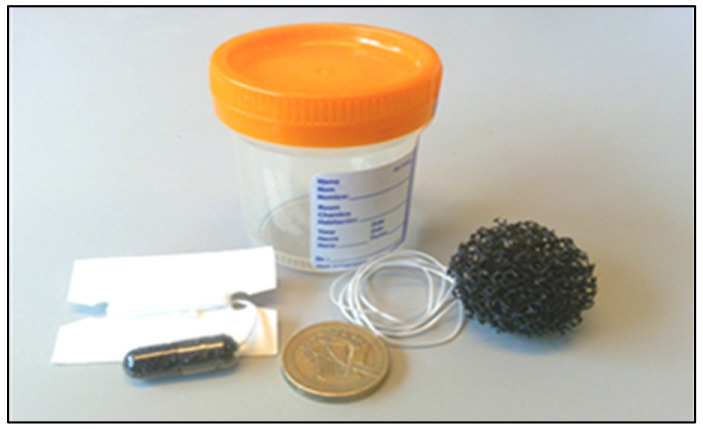
Cytosponge™ capsule with string is swallowed with 150 mL of water. After 5 min, the gelatin coat of the capsule will be dissolved, and the sponge that is inside the capsule will be released. The sponge gathers cells from the esophagus and gastroesophageal junction upon retrieval by pulling the string. The retrieved and expanded sponge is placed in a preservative fluid and transferred to the laboratory for cytological examination and staining.

Recent studies have shown that Barrett’s neoplasia can be missed during routine upper gastrointestinal endoscopy by many endoscopists in primary and secondary gastrointestinal care centers [257]. Further, most patients diagnosed with EAC have no prior history of GERD or diagnosis of Barrett’s esophagus, which demonstrates the failure of current screening practices [258,259]. Studies suggest that patients with EAC who do not have coexisting Barret’s esophagus at the time of cancer diagnosis may have a more aggressive form of EAC with a poorer prognosis compared to patients with Barret’s esophagus associated EAC [259]. Consequently, the present screening guidelines have limited ability to detect prevalent early ESCC and EAC [259]. Because symptomatic presentation remains the predominant route to EC diagnosis, there is a growing interest and need to develop techniques to detect the disease at an early curable stage [257]. More optimized screening methods that do not rely on GERD symptoms are needed to identify EC precursor lesions in high-risk populations [258]. Endoscopic surveillance of high-risk populations is expensive and requires specialized settings that are not accessible for most high-risk individuals, particularly those who live in developing regions. Efforts are required to develop less invasive, less expensive, and more implementable screening methods and biomarker assays for the early detection of both EAC and ESCC and their precursor lesions. There has been immense progress in the UK in the combination of novel, less-invasive, safe, and affordable methods for EC screening and early diagnosis in high-risk groups. These methods include biomarker assays applied to esophageal cytological collections obtained using less-invasive methods, such as the Cytosponge™ (Figure 4). This pill on a string has been shown to be feasible and as effective as endoscopic screening both for EAC and (in fewer patients) ESCC and could be used to screen high-risk patients who do not have GERD symptoms [260,261,262].

A randomized controlled trial in the United Kingdom showed that the use of the Cytosponge™ in general practice clinics to screen individuals with GERD symptoms who were receiving PPI treatment was associated with a 10-fold higher detection of Barret’s esophagus compared with the standard care [260]. The Cytosponge™ has also been found to be a feasible, safe, and acceptable screening method for early diagnosis of high-grade esophageal squamous dysplasia with promising initial accuracy data in Northeast Iran, a high-risk area for ESCC [261].

## 5. Diagnosis

Having a positive history of tobacco smoking (and/or opium smoking in some regions), alcohol drinking, and low SES are more common among patients with ESCC [263], while having higher BMI, positive history of smoking, and having GERD and higher SES are more common among patients with EAC [264]. While the clinical and demographical features could raise clinical suspicion about EC and its subtype, the diagnosis should be confirmed by upper gastrointestinal endoscopy with biopsy. Endoscopic ultrasound (EUS) is the standard technique to establish the locoregional stage and to guide the therapeutic plan, particularly for patients who do not present with distant metastases [265]. EUS also provides the possibility of biopsy in case of noticing a suspicious lymph node. Computed tomography (CT) scan or magnetic resonance imaging (MRI) should also be performed to investigate the presence of distant metastasis and to guide treatment plans [2]. MRI is also usually used for detecting locally advanced diseases and surveillance after neoadjuvant therapy.

A wide range of biomarkers, including immunohistochemical markers, blood-based markers, MicroRNA markers, long noncoding RNAs, and DNA-based markers, have been identified in various studies which were suggested to have diagnostic potential for detecting ESCC and EAC [266,267,268,269,270]. However, each of these biomarkers has its limitations, and none have been translated into effective clinical tools [266,267,268,269,270]. Other limitations of the current biomarker research for the diagnosis of EC include small sample size, research design of a single population, lack of value for early diagnosis, lack of independent verification studies, and lack of pre-clinical data [266,267,268]. In addition to the requirement for more technologies and detection methods to settle the problem of the low abundance of some biomarkers in biological samples and further enhance the quality of detection, studies can focus on building combined detection methods to gain better diagnostic value. Finally, more clinical studies are needed to identify the appropriate collection methods and cutoff values for the identified biomarkers for the detection of EC [266,267,268].

## 6. Treatment

### 6.1. Current Treatment Strategies

Despite the advances in therapeutic strategies and the improvement in EC survival rates over the past two decades, EC still has a poor prognosis with an overall five-year survival of less than 20% [6,7]. Although ESCC and ESC are biologically different and have different molecular profiles, there is still a considerable overlap in the treatment strategies for these two EC subtypes. Locally advanced disease is treated for cure with multi-modality therapy, while the advanced disease is treated with palliative purpose [271]. EC treatment is usually associated with a significant decline in health-related quality of life [2,263]. 

Endoscopic eradication therapy (EET) is the best strategy for treating premalignant lesions and early-stage EC when there is no evidence of lymph node involvement [246]. Recent studies have shown comparable two- and five-year survival rates among patients with early EC who underwent EET and surgical resection [272,273]. The selection of endoscopic mucosal resection (EMR) versus endoscopic submucosal dissection (ESD) is controversial and should be based on the physician’s expertise and the lesions’ characteristics, including their size [271]. Two recent meta-analyses showed higher en bloc, curative, and R0 resection rates and lower recurrence rates in ESD compared with EMR [274,275]. However, ESD was significantly more time-consuming and induced more perforations than the EMR procedure [275]. 

Curative treatment for advanced EC typically includes chemotherapy or chemoradiotherapy followed by extensive surgery, which often results in considerable morbidity and persistent reductions in health-related quality of life [264]. ESCC is more responsive to chemoradiotherapy, and its endoscopic response rates are higher than EAC [276]. Therefore, in contrast to patients with EAC, it is possible to reserve the esophagus after chemoradiotherapy in patients with ESCC [276]. A recent review of clinical trials concluded that adding esophagectomy to chemoradiotherapy in locally advanced ESCC provides little or no difference in overall survival and may be associated with higher treatment-related mortality [277,278]. When esophagectomy is indicated, minimally invasive esophagectomy (MIE) and robot-assisted minimal invasive esophagectomy (RAMIE) can be done safely with the enhanced postoperative outcome and similar oncological results compared with open esophagectomy [279].

In patients with early EAC (stage T1a) or superficial pT1b lesions that are less than 3 cm and have well-differentiated histology with no lymphovascular invasion, ESD could be the treatment of choice [280,281,282]. However, more studies are needed to assess the long-term outcomes after endoscopic resection of pT1b tumors. Surgery is the alternative treatment to cure patients with early EAC with localized tumors. Patients with EAC who refuse endoscopic therapy or have more advanced diseases should also be considered for esophagectomy. Local recurrence usually occurs within one year after endoscopic resection [283]. Therefore, surveillance endoscopy is recommended for all patients who undergo endoscopic therapy, which can be done every three months for the first year, then every four–six months for the next year, and then annually.

For advanced EAC, surgery alone is not enough, and preoperative chemoradiation should be considered in addition to surgery. Recently, immunotherapy has revolutionized EC treatment. Several large clinical trials showed promising results for immunotherapy with high efficacy, tolerable toxicity, improved survival rates, and better quality of life [284,285].

### 6.2. Future Perspectives for Treatment of Esophageal Cancer

We still need novel treatments to improve the survival and quality of life in patients with EC. Immunotherapy and targeted therapy are rapidly evolving at the forefront of new treatments for the EC [286,287]. Further, rapidly improving knowledge of the genomics and tumor microenvironment of EC offers promising opportunities for further treatment discoveries [286]. Therefore, of the main challenges for the future is to identify molecular targets based on tumor profiling that could help tailor treatment regimens and move toward personalized treatment strategies [288]. Information on the status of human epidermal growth factor receptor 2 (HER2), expression of programmed cell death protein 1 (PD-L1), microsatellite instability (MSI), and mutational burden of EC tumors may become increasingly relevant for the incorporation of targeted therapy within therapeutic strategies, and to help to monitor the response to treatments [288].

Recent genetic studies of EC tumors identified distinctive mutational signatures and molecular patterns for each of the ESCC and EAC subtypes that may have potential therapeutic relevance [47,289]. Furthermore, the Cancer Genome Atlas (TCGA) reported an integrated genomic landscape in EAC and ESCC, which can be utilized to identify potential therapeutic targets for EC subtypes [290]. Examples of potential therapeutic targets that were suggested by recent studies include the enzyme nicotinamide N-methyltransferase (NNMT), which is upregulated in EC tumors and contributes to their aggressiveness [291], and the long noncoding RNAs (lncRNAs) that regulate the development and progression of multiple cancers, including EC [292]. A number of NNMT inhibitors are already available; therefore, the effectiveness of these agents in treating EC could be assessed in appropriate studies [293,294].

Finally, drug repositioning has been recently suggested as a feasible and cost-effective strategy to identify novel treatments for EC [295]. This approach is based on identifying existing drugs that have already been approved for treating other conditions, which can be repurposed to treat patients with EC [295]. In this approach, the gene expression profiles of disease states are compared with the effect on gene expression by a given drug [295].

## Figures and Tables

**Table 1:** Environmental risk factors for esophageal cancer (based on human studies).

Habits	
Tobacco	Causal association with EC recognized by IARC monograph evaluation (Group 1) (for both smoking and smokeless tobacco) Strong association with ESCC (3–9 fold risk increase) Moderate association with EAC (2–3 fold risk increase)
Alcohol	Causal association with EC recognized by IARC monograph evaluation (Group 1) Strong association with ESCC for very heavy intake (around 5-fold risk increase) No association with EAC
Opium	Mild to moderate association with ESCC (1.6 to 2-fold risk increase) No studies on EAC
Hot food and drinks	Moderate association with ESCC (around 2- to 3-fold risk increase) IARC monograph evaluation: probably carcinogenic (Group 2A) No association with EAC
**Living environment**	
Socioeconomic status	Mild to moderate association with ESCC (1.5 to 2-fold risk increase) Mild to moderate association with EAC (1.5 to 2-fold risk increase)
Household fuel	Using biomass: moderate association with ESCC (around 2-fold risk increase) No studies on EAC
Water source	Drinking un-piped water: moderate association with ESCC (around 2-fold risk increase) No studies on EAC
Mycotoxin contamination	Inconsistent association with ESCC No studies on EAC
Animal contact	Inconsistent association with ESCC No studies on EAC
**Individual health**	
Gastroesophageal reflux disease (GERD)	Strong association with EAC (5-9 fold risk increase) Inconsistent association with ESCC
Body mass index (BMI)	Mild to moderate inverse association with ESCC (around 30% risk decrease) Mild to moderate association with EAC (1.5 to 2-fold risk increase)
Gastric atrophy	Moderate association with ESCC (2-fold risk increase) No association with EAC
Poor oral health and oral hygiene	Lack of tooth brushing: moderate association with ESCC (2-fold risk increase) Tooth loss: mild to moderate association with ESCC and EAC (1.3 to 2-fold risk increase) Periodontitis: mild association with ESCC and EAC (1.4-fold risk increase)
Microbiome	Composition of specific oral and esophageal bacterial communities, lower oral microbial diversity, and lower esophageal microbial richness may be linked to ESCC Changes in the ratio of specific Gram-negative esophageal bacteria may be linked to Barrett’s esophagus (EAC precursor lesion) No clear, consistent pattern in relation to EAC
**Dietary factors**	
Whole diet quality	Better diet quality linked to lower EC risk Adherence to plant-based dietary patterns (Mediterranean diet) is associated with lower ESCC risk Adherence to WCRF–AICR recommendations that include diet, physical activity, and BMI is associated with lower EAC risk
Intake of specific dietary groups	Higher intake of fruits and vegetables: strong inverse association (45% risk decrease) with ESCC and moderate inverse association (25% risk decrease) with EAC Higher intake of pickled vegetables: moderate association with ESCC (2-fold risk increase) Higher intake of red and processed meat: mild association (30% to 70% risk increase) with both ESCC and EAC Higher intake of carbohydrates: strong inverse association (40% risk decrease) with ESCC and EAC Higher intake of dietary fats: mild association with EAC (70% risk increase) and no association with ESCC Higher intake of poultry and fish: inconsistent results for ESCC and no association with EAC Higher consumption of dairy products: no association with ESCC or EAC
Intake of micronutrients	Interventional studies and meta-analyses showed no effects for vitamin and antioxidant supplements in preventing esophageal cancer, but a possible protective effect if supplementation occurred in a younger adult Inconclusive results for selenium and zinc deficiency and ESCC risk
**Infections**	
Viral infection	Human papilloma virus (HPV): no association for most HPV subtypes. Positive association may be present for rare subtypes. Other viruses: inconsistent associations with EC
Bacterial infection	Helicobacter pylori: strong inverse association with EAC (50% risk decrease) No association with ESCC

**Table footnotes: IARC:** International Agency for Research on Cancer; **EC:** esophageal cancer; **ESCC:** esophageal squamous cell carcinoma; **EAC:** esophageal adenocarcinoma; **WCRF–AICR**: World Cancer Research Fund–American Institute for Cancer Research.
